# *Dyella cornirhiza* sp. nov., a Maize Rhizosphere Bacterium with Diverse Biosynthetic Potential

**DOI:** 10.3390/microorganisms14091943

**Published:** 2026-09-02

**Authors:** Ying Li, Lei Shen, Miaomiao An, Xuming Wang, Guozhu Zhao, Tianlei Qiu

**Affiliations:** 1 National Engineering Research Center of Tree Breeding and Ecological Restoration, College of Biological Sciences and Technology, Beijing Forestry University, Beijing 100083, China; ly19833830219@163.com (Y.L.); anmiao315418@163.com (M.A.); 2 Beijing Key Laboratory of Agricultural Genetic Resources and Biotechnology, Institute of Biotechnology, Beijing Academy of Agriculture and Forestry Sciences, Beijing 100097, China; wangxuming@baafs.net.cn; 3 College of Life Sciences, Langfang Normal University, Langfang 065000, China; shenlei@lfnu.edu.cn

**Keywords:** *Dyella*, pan-genome analysis, biosynthetic gene clusters, saframycin, maize soil, novel bacterial species

## Abstract

A novel bacterial strain, CR191^T^, was isolated from maize (*Zea mays*) rhizosphere soil. The strain is Gram-stain-negative, oxidase-positive, rod-shaped, non-motile, and aerobic. Phylogenetic analysis based on 16S rRNA gene sequences placed CR191^T^ within the genus *Dyella*, with highest similarity to *Dyella flava* DHOC52^T^ (98.23%) and *Dyella dinghuensis* DHOA06^T^ (98.16%). The genomic DNA G+C content was 66.19%. Average nucleotide identity (ANI) and digital DNA-DNA hybridization (dDDH) values between strain CR191^T^ and the type strains of the other 36 validly published *Dyella* species ranged from 79.0 to 86.6% and 20.2–28.7%, respectively, both below the recognized thresholds for species delineation. Genome mining identified several putative biosynthetic gene clusters showing similarity to known clusters associated with diverse secondary metabolites, including saframycin, cosmomycin, and malleobactin biosynthesis. The major cellular fatty acids were iso-C_17:0_, iso-C_15:0_, and summed feature 9 (comprising iso-C_17:1_ ω9c and/or C_16:0_ 10-methyl). The polar lipid profile comprised phosphatidylglycerol, phosphatidylethanolamine, phosphatidylmonomethylethanolamine and two unidentified polar lipids; the sole respiratory quinone was ubiquinone Q-8. Based on a combination of phenotypic, chemotaxonomic, genomic, and phylogenetic characteristics, strain CR191^T^ is considered to represent a novel species of the genus *Dyella*, for which the name *Dyella cornirhiza* sp. nov. is proposed. The type strain is CR191^T^ (=CCAM 2032^T^ = JCM 36807^T^).

## 1. Introduction

The rhizosphere, a critical interface between plant roots and soil, harbors a diverse and functionally complex microbial community that profoundly influences plant growth, health, and stress tolerance [1]. Understanding the taxonomic and functional diversity of these root-associated bacteria is essential for unraveling plant-microbe interactions and harnessing their potential for sustainable agriculture. Within this niche, members of the genus *Dyella* (family *Rhodanobacteraceae*, class *Gammaproteobacteria*) have attracted increasing attention because of their broad ecological distribution and metabolic versatility [2].

The genus *Dyella* was first proposed in 2005 with *Dyella japonica* as the type species [2]. As of July 2026, the genus comprises 36 validly published species (https://lpsn.dsmz.de/genus/dyella, accessed on 1 July 2026). Members of the genus have been isolated predominantly from terrestrial environments, including forest [3,4], grassland soils [5], rhizosphere soils [6,7], cliff soils [8], and other specialized habitats such as rock surfaces [9] and winery sediments [10], reflecting substantial ecological diversification within the genus. *Dyella* species are generally characterized as Gram-stain-negative, aerobic, rod-shaped bacteria with ubiquinone-8 (Q-8) as the predominant respiratory quinone [7]. Recent studies have revealed substantial functional diversity within the genus *Dyella*. For example, *D. thiooxydans* [6] is capable of oxidizing thiosulfate, indicating a potential role in sulfur cycling. In addition, *Dyella ginsengisoli* LA-4 [11] can utilize biphenyl as the sole carbon source and efficiently degrade high concentrations of biphenyl. Notably, *Dyella thailandensis* [12] exhibited strong potential for lignocellulose degradation. *Dyella* species have also been reported to degrade triclosan [13] and produce industrially important enzymes such as isoamylase [14]. However, the biosynthetic gene cluster repertoire of the genus *Dyella* remains largely unexplored, particularly with respect to its potential for secondary metabolite biosynthesis. Therefore, the broad ecological distribution and metabolic diversity of *Dyella* species have attracted increasing attention.

Despite these insights, the diversity of *Dyella* within specific agricultural ecosystems, particularly the rhizosphere of globally significant crops like maize (*Zea mays*), remains underexplored. Moreover, while recent genomic studies have begun to reveal the biosynthetic potential of various bacterial genera, the diversity of secondary metabolite biosynthetic gene clusters (BGCs) in *Dyella* remains poorly characterized. In particular, the occurrence of BGCs showing similarity to known secondary metabolite biosynthetic pathways in this genus warrants further investigation. This gap in genomic knowledge limits our understanding of the metabolic potential of *Dyella* and its possible relevance in agricultural environments.

Strain CR191^T^ was isolated from maize rhizosphere soil in Sanya, China. Its 16S rRNA gene sequence suggested affiliation with the genus *Dyella* as a potentially novel species. To confirm this, we performed a comprehensive polyphasic study integrating phenotypic, chemotaxonomic, phylogenetic, and genomic analyses. Beyond taxonomic characterization, we analyzed the genome for functional features, with particular emphasis on secondary metabolite BGCs and carbon metabolic pathways. This work provides genomic insights into the metabolic potential of strain CR191^T^, a maize rhizosphere-associated *Dyella* strain.

## 2. Materials and Methods

### 2.1. Isolation and Ecology

Strain CR191^T^ was isolated from the rhizosphere soil of maize (*Zea mays*) cultivated in Sanya City, Hainan Province, China (18°23′24″ N, 109°09′32″ E). For rhizosphere soil collection, maize plants were carefully excavated, and loosely adhering soil was gently removed from the roots. The soil remaining tightly attached to the root surface was collected as rhizosphere soil and used for bacterial isolation. To isolate culturable bacteria, a soil sample was subjected to serial dilution (10^−2^ to 10^−6^) in sterile 0.85% (*w*/*v*) saline and plated onto Reasoner’s 2A (R2A) agar. Plates were incubated aerobically at 30 °C for 7 days. Distinct colonies were selected based on morphological variation and purified through three successive streaks on fresh R2A agar. Pure cultures were preserved at −80 °C in R2A broth supplemented with 20% (*v/v*) glycerol. Preliminary 16S rRNA gene sequence analysis indicated that strain CR191^T^ represents a putative novel species within the genus *Dyella*. Consequently, the strain was deposited in two public culture collections: the China Collection of Anaerobic Microorganisms (CCAM) and the Japan Collection of Microorganisms (JCM), under accession numbers CCAM 2032^T^ and JCM 36807^T^, respectively. For comparative analysis, the reference type strains *Dyella flava* KCTC 52128^T^ and *Dyella dinghuensis* KCTC 52129^T^ were obtained from Korean Collection for Type Cultures (KCTC), while *Dyella nitratireducens* CGMCC 1.15439^T^ was sourced from China General Microbiological Culture Collection Center (CGMCC).

### 2.2. 16S rRNA Gene-Based Phylogenetic Analysis

Genomic DNA was extracted using a standard protocol, and the nearly full-length 16S rRNA gene was amplified with the universal bacterial primers 27F and 1492R [15]. The resulting PCR product was cloned into the pClone007 vector using the 5min TA/Blunt-Zero Cloning Kit (Beijing Tsingke Biotech Co., Ltd., Beijing, China), and the recombinant clone was subsequently sequenced using the M13F and M13R primers. The obtained 16S rRNA gene sequence of strain CR191^T^ was compared against the EzBioCloud database [16] and subjected to BLASTN searches against the National Center for Biotechnology Information (NCBI) nucleotide database (https://www.ncbi.nlm.nih.gov/). Multiple sequence alignment of representative *Dyella* sequences was performed, and phylogenetic trees were reconstructed with three independent algorithms: neighbor-joining (NJ) [17], minimum-evolution (ME) [18] and maximum-likelihood (ML) [19], all implemented in MEGA 11 [20]. Genetic distances were calculated using the Kimura two-parameter model [21], and branch support was assessed by bootstrap analysis with 1000 replicates [22].

### 2.3. Genome Sequencing and Analysis

The genome of strain CR191^T^ was sequenced using a hybrid approach combining Oxford Nanopore PromethION (ONT, Oxford, UK) and Illumina NovaSeq 6000 platforms (Benagen, Wuhan, China). De novo assembly was performed with Unicycler v0.5.0 [23]. Gene prediction and annotation were carried out using Prokka v1.14.6 [24]. Functional annotation of protein-coding genes was assigned based on the following databases: Gene Ontology (GO) [25], Clusters of Orthologous Groups (COG) [26], Kyoto Encyclopedia of Genes and Genomes (KEGG) [27] and carbohydrate-active enzymes database (CAZy) [28]. Putative BGCs for secondary metabolites were identified with antiSMASH v6.0.0 [29]. The genomic DNA G+C content was calculated directly from the assembled sequence. Average nucleotide identity (ANI) values were estimated using fastANI v1.34 (https://github.com/ParBLiSS/FastANI, accessed on 1 July 2026) [30]. The digital DNA-DNA hybridization (dDDH) values were computed with the Genome-to-Genome Distance Calculator (GGDC 3.0; http://ggdc.dsmz.de/ggdc.php, accessed on 1 July 2026) based on Formula 2 (identities/HSP length) [31]. Antibiotic resistance genes were screened against the Comprehensive Antibiotic Resistance Database (CARD; http://arpcard.mcmaster.ca, accessed on 1 July 2026) [32]. Additional functional annotation was performed using eggNOG-mapper 5.0 (http://eggnog-mapper.embl.de/, accessed on 1 July 2026) [33]. Phylogenomic analyses were conducted with the Up-to-date Bacterial Core Gene (UBCG) pipeline [34] based on 92 conserved core genes.

Metabolism estimation and pan-genomic analysis were conducted and visualized using the Anvi’o pan-genome workflow (version 6) [35]. The FASTA files were reformatted using anvi-script-reformat-fasta and imported as contigs-DB with anvi-gen-contigs-database. Gene calling was performed with anvi-run-prodigal, and functional annotation was carried out using anvi-run-pfams.

### 2.4. Phenotypic and Physiological Characterization

Cell morphology was observed via transmission electron microscopy (HT7800, Hitachi, Tokyo, Japan) after 48h of growth in R2A agar at 30 °C. Gram staining was performed following the manufacturer’s protocol (Solarbio Gram staining kit, Beijing Solarbio Science & Technology Co., Ltd., Beijing, China). Growth temperature range and optimum were assessed in R2A broth at 4, 10, 15, 20, 25, 28, 30, 37, 42, and 50 °C, with growth quantified by measuring the OD600. The pH range for growth was determined between pH 2.0 and 12.0 (increments of 1 pH unit) using the buffer system described by Xu et al. [36], and growth was quantified by measuring the OD600. Salt tolerance was evaluated in R2A broth supplemented with various concentrations (0, 1, 2, 3, and 5%, *w*/*v*) NaCl, with growth quantified by measuring the OD600. All growth assays were performed in triplicate. Catalase activity was detected by bubble formation in 3% (*v*/*v*) H_2_O_2_, and oxidase activity was tested with commercial oxidase reagent (bioMérieux, Marcy-l’Étoile, France). Additional biochemical properties and enzyme activities were profiled using the API 20NE, API 50CH and API ZYM kits (bioMérieux), while carbon source utilization was examined with GEN III MicroPlates (Biolog, Inc., Hayward, CA, USA), all according to the manufacturer’s instructions. Antibiotic susceptibility was determined by the disk diffusion method [37] on R2A agar at 28 °C with filterpaper disks (Oxoid, Basingstoke, UK) containing the following antibiotics (concentration per disk): norfloxacin (10 µg), carbenicillin (100 µg), cefoxitin (30 µg), streptomycin (300 µg), tetracycline (30 µg), clindamycin (2 µg), levofloxacin (5 µg), erythromycin (15 µg), gentamicin (10 µg), trimethoprim/sulfamethoxazole (25 µg), penicillin G (10 IU), vancomycin (30 µg), neomycin (30 µg), amikacin (30 µg), kanamycin (30 µg), ciprofloxacin (5 µg), ceftriaxone (30 µg), ampicillin (2 µg), cefoperazone (30 µg), enrofloxacin (5 µg), florfenicol (30 µg), polymyxin B (300 IU), and rifampicin (5 µg). The bacterial suspension was adjusted to an OD600 of 0.5 before inoculation. Inhibition-zone diameters were measured after 48 h of incubation. Because standardized antimicrobial susceptibility breakpoints are not available for *Dyella*, inhibition-zone diameters were used for comparative phenotypic characterization.

### 2.5. Chemotaxonomic Analysis

For fatty acid analysis, CR191^T^ and the reference strains *D. flava* DHOC52^T^, *D. dinghuensis* DHOA06^T^ and *D. nitratireducens* DHG59^T^ were grown on R2A medium at 30 C for 48 h. Cells were harvested under standardized conditions and subjected directly to fatty acid extraction according to the standard protocol of the MIDI system (Sherlock Microbial Identification System, version 6.2B). Fatty acid methyl esters were analyzed using an Agilent 8860 gas chromatograph and identified with the TSBA6 database of the Microbial Identification System [38]. To analyze the polar lipid and quinone profiles of strain CR191^T^, cells were cultured in R2A broth at 30 °C for 48 h in a shaking incubator. Polar lipids were extracted and separated by two-dimensional TLC following the method of Minnikin et al. [39]. Respiratory quinones were extracted with methanol/chloroform, followed by solvent partitioning and collection of the methanol phase for subsequent analysis by high-performance liquid chromatography (HPLC) [40,41].

## 3. Results and Discussion

### 3.1. Phylogenetic Analysis Based on 16S rRNA Gene Sequence

**Figure 1:**
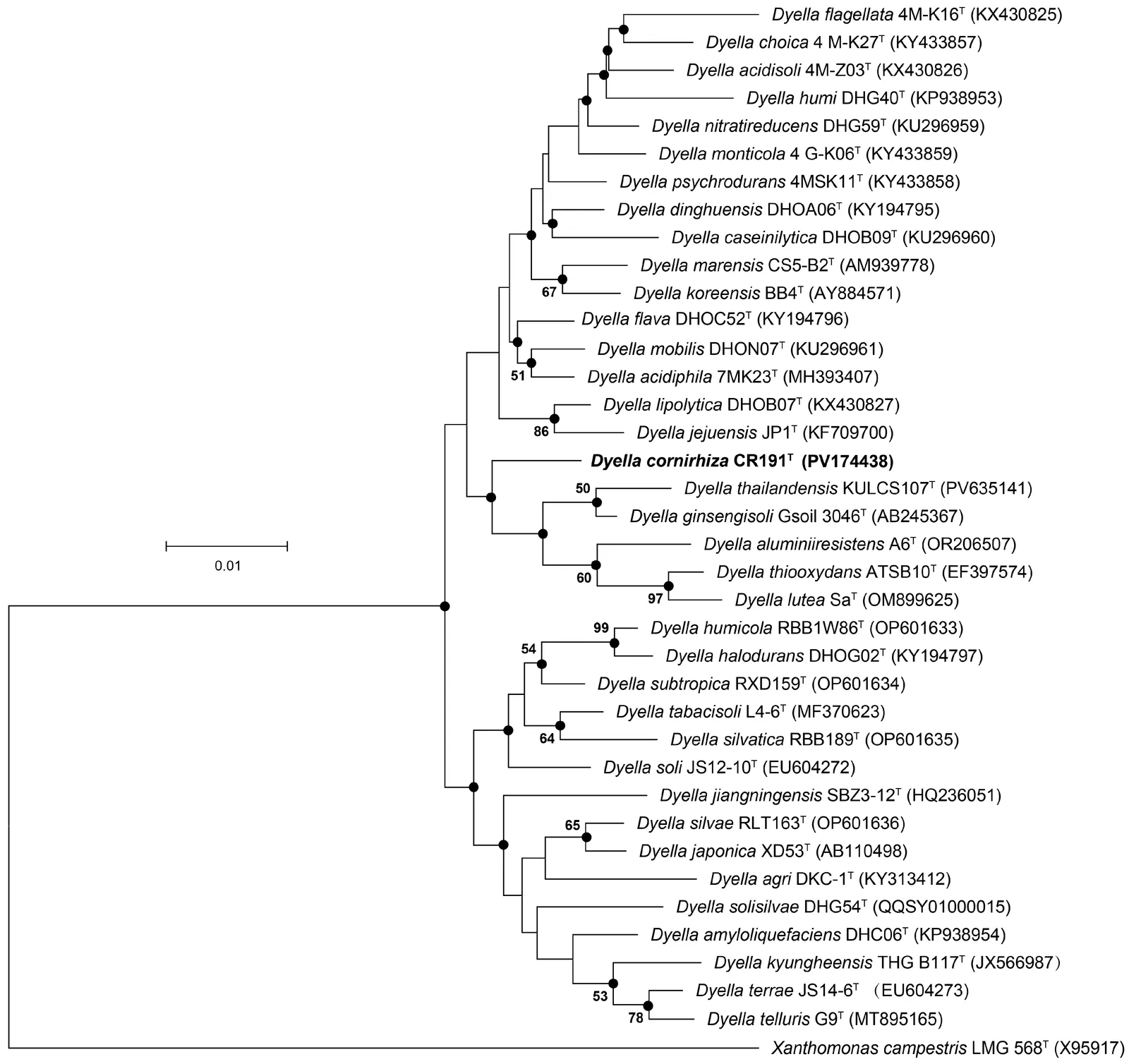
Phylogenetic tree reconstructed using the neighbor-joining method based on 16S rRNA gene sequences of strain CR191^T^ and species of the genus *Dyella*. *Xanthomonas campestris* LMG 568^T^ (X95917) was used as the outgroup. Numbers at branch nodes represent confidence levels (values ≥ 50% are shown) based on 1000 replicates bootstrap samplings. GenBank accession numbers are given in parentheses. Bar, 0.01, represents the number of substitutions per nucleotide site. Filled cycles indicates branches that are also found in maximum-likelihood and minimum-evolution trees.

The 16S rRNA gene sequence of strain CR191^T^ was 1506 bp in length and was deposited in the GenBank database (accession number: PV174438). Comparative analysis using EzBioCloud/EzTaxon-e server revealed that strain CR191^T^ is phylogenetically affiliated with the genus *Dyella*, showing the highest sequence similarity to *Dyella flava* DHOC52^T^ (98.23%) [42], followed by *Dyella dinghuensis* DHOA06^T^ (98.16%) [43] and *Dyella nitratireducens* DHG59^T^ (98.15%) [44]. The 16S rRNA gene sequence similarities (98.15–98.23%) were below the recommended threshold range (98.7%) for species delineation, but alone are insufficient for conclusive taxonomic assignment, necessitating whole-genome-based analyses [45]. Phylogenetic reconstruction further demonstrated that strain CR191^T^ forms a distinct lineage within the genus *Dyella* (Figure 1), supporting its status as a novel species.

### 3.2. Genome Analysis

**Figure 2:**
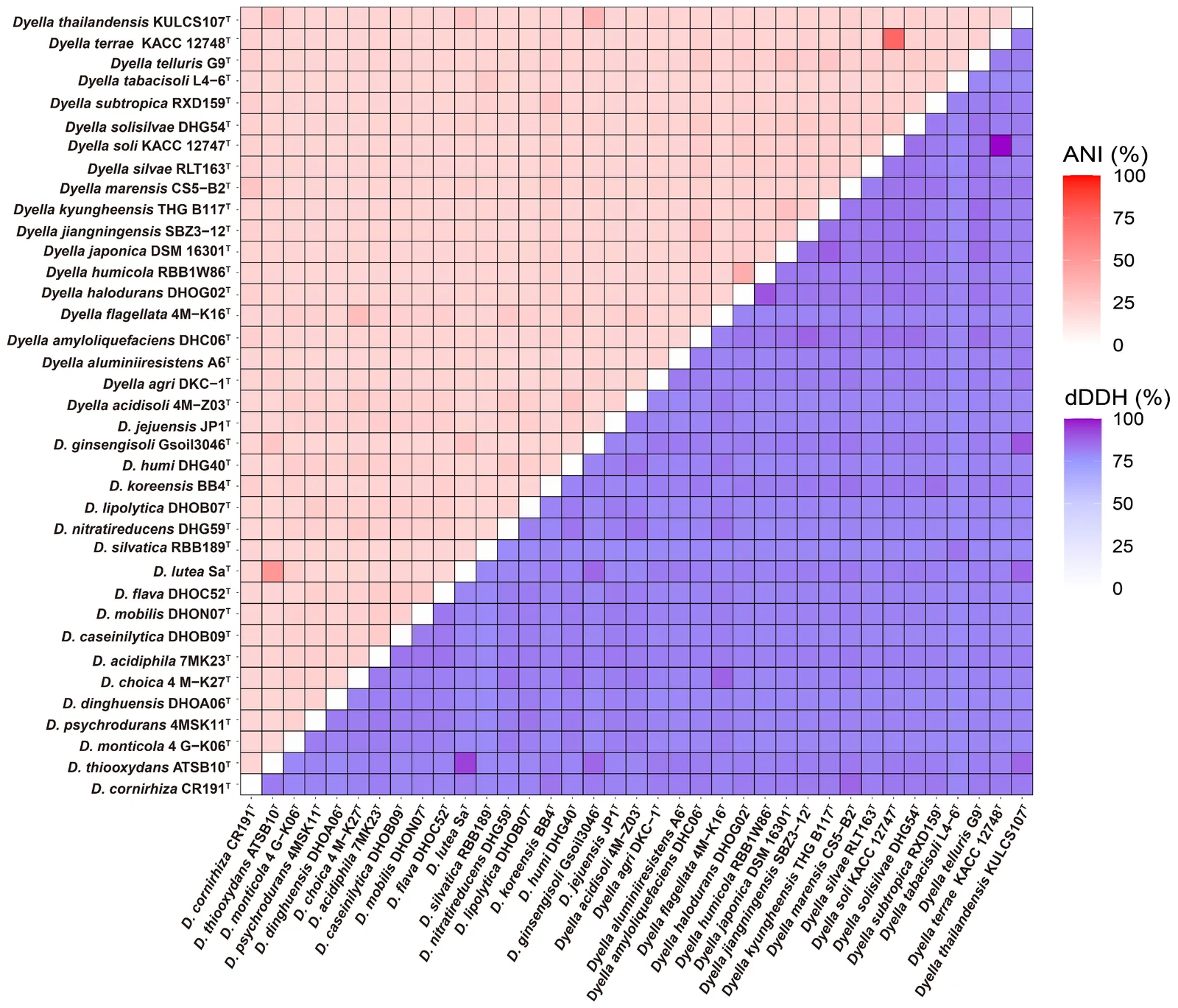
Heatmap matrix of average nucleotide identity (ANI) and digital DNA-DNA hybridization (dDDH) values for 37 *Dyella* species. Pairwise genome comparisons are displayed in a square matrix divided diagonally: the upper triangle presents ANI values in shades of red, while the lower triangle shows dDDH values in shades of purple. Color intensity reflects similarity levels, with darker shades indicating higher values.

The whole-genome sequencing of strain CR191^T^ generated an average coverage depth of 333×. The final assembly comprises 5,210,872 bp with an N50 value of 18,779 bp and a DNA G+C content of 66.19%. A total of 4615 genes were predicted, mainly including 4535 protein-encoding genes, 55 tRNA genes, and 5 rRNA genes (5S, 16S, 23S). The 16S rRNA gene sequence extracted from the genome was identical to that obtained by PCR amplification, confirming the integrity of the assembly [46]. Comparative genomic analysis revealed that strain CR191^T^ shared ANI values ranging from 79.0 to 86.6% and dDDH values ranging from 20.2 to 28.7% with the type strains of the other 36 validly published *Dyella* species. ANI and dDDH values for 37 *Dyella* species are visualized in the ANI/dDDH heatmap (Figure 2). Both ANI and dDDH values are well below the established thresholds for species delineation (95–96% for ANI and 70% for dDDH) [47,48]. A comprehensive comparison of ANI and dDDH values between the novel strain CR191^T^ and 36 *Dyella* species is presented in the attached Supplementary Excel S1, further supporting the genomic distinctiveness of strain CR191^T^ from all other currently recognized *Dyella* species.

**Figure 3:**
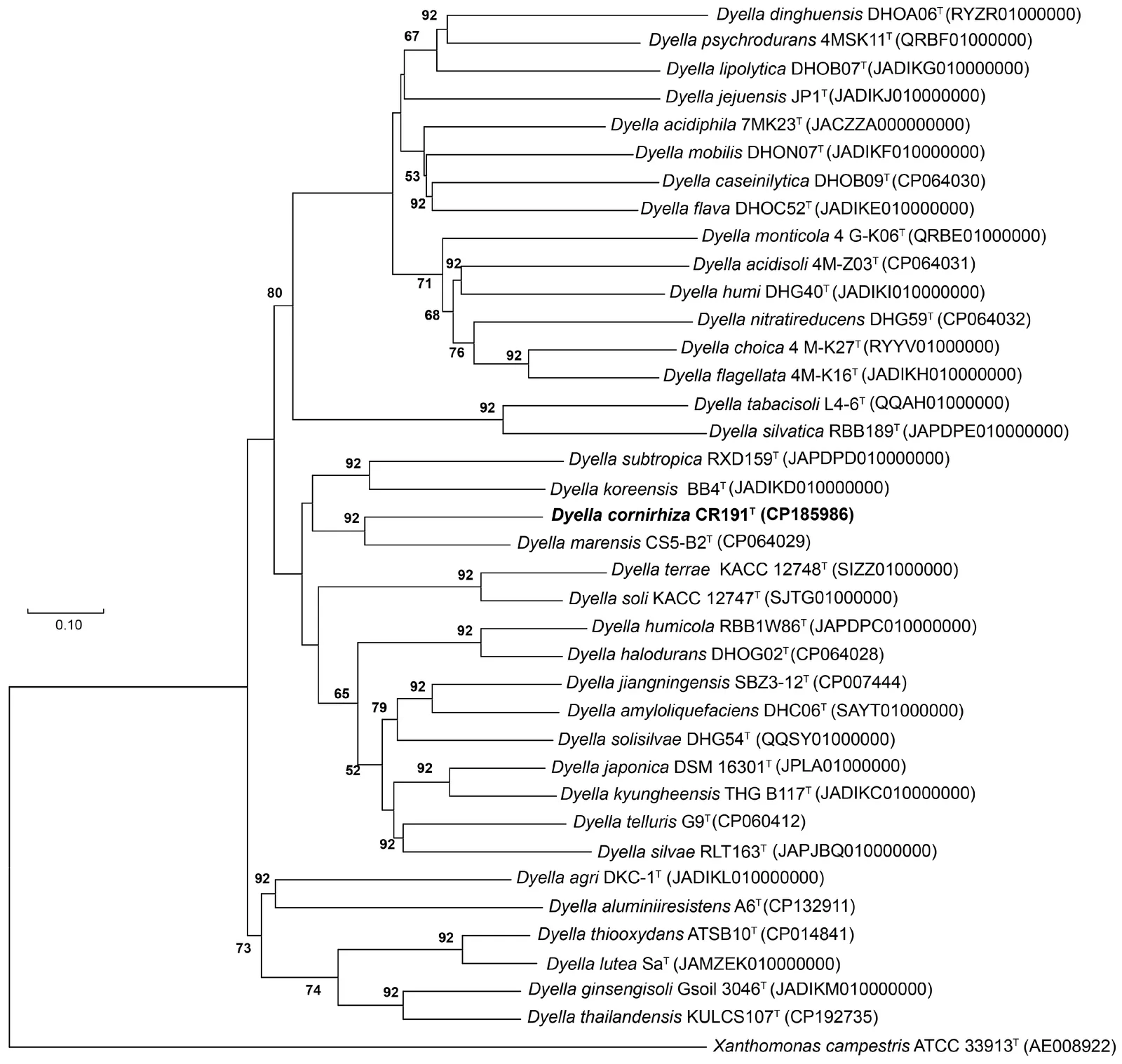
Maximum-likelihood phylogenomic tree based on 92 conserved bacterial core genes showing the phylogenetic position of strain CR191^T^ among the type strains of the genus *Dyella*. Gene Support Index (GSI) values greater than 50 are shown at the branch nodes. *Xanthomonas campestris* ATCC 33913^T^ was used as the outgroup. Whole-genome GenBank accession numbers are given in parentheses. The scale bar represents 0.10 substitutions per nucleotide position.

The Up-to-date Bacterial Core Gene (UBCG) pipeline provides a standardized approach for reconstructing bacterial phylogenetic relationships based on a set of conserved single-copy core genes [34]. The original UBCG framework comprises 92 bacterial core genes, which are selected based on their broad distribution and single-copy occurrence across bacterial genomes. In this study, the UBCG approach was used to assess the phylogenetic position of strain CR191^T^ and its related *Dyella* species. The resulting tree, constructed from a concatenated alignment of these core genes, placed CR191^T^ within the genus *Dyella*, forming a distinct lineage sister to *D. marensis* CS5-B2^T^ (Figure 3). This result corroborates the affiliation of CR191^T^ with the genus *Dyella* while highlighting its phylogenetic distinctiveness.

**Figure 4:**
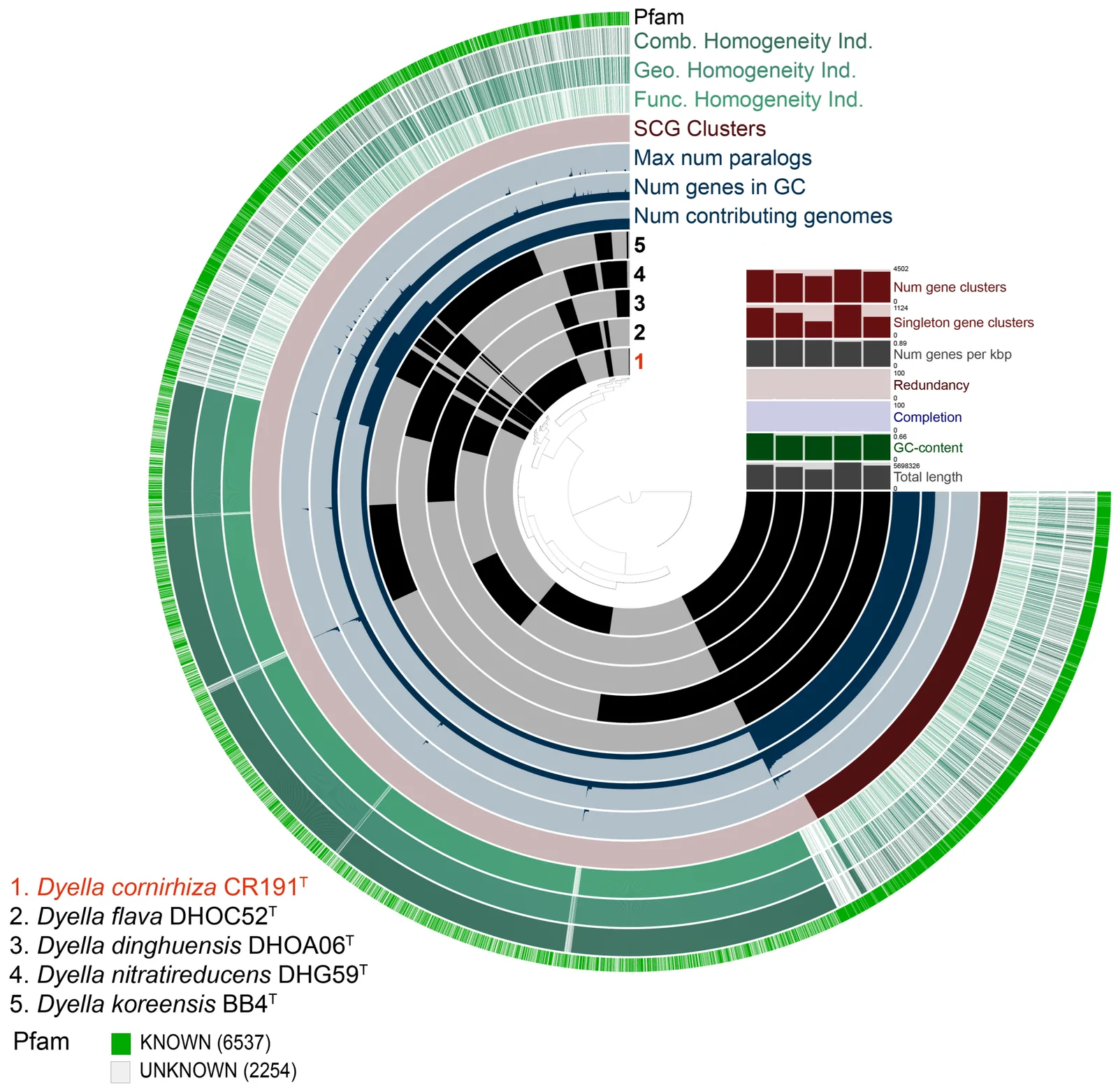
Pan-genome analysis of four reference strains and the novel strain *Dyella cornirhiza* CR191^T^. Genomes are clustered based on the presence/absence of 8791 gene clusters, illustrating shared and unique genomic content across strains.

The Anvi’o workflow was employed to validate the core genome and pan-genome structures of strain CR191^T^ and four reference strains. The resulting pan-genome comprised 8791 gene clusters, including 2070 core genes and 4248 singletons (genes unique to individual genomes). Here, the core gene clusters represent genes shared among the five genomes analyzed, whereas the singleton gene clusters represent genes occurring in only one of the five genomes. Quantitatively, core gene clusters accounted for 23.5% of the entire pan-genome, whereas singleton gene clusters represented 48.3%, indicating considerable genomic diversity among the five *Dyella* genomes examined. The inner edge of the pan-genome visualization illustrates the number of genomes containing specific gene clusters, the number of genes within those clusters, and the maximum number of paralogs (navy). It also includes geometric, functional and combined homogeneity indices (green), as well as the distribution of single-copy gene clusters (brown). The outer layers represent total genome length, GC content, genome completeness, redundancy, gene density (genes per kbp), number of singleton gene clusters and total gene cluster count (Figure 4).

Functional annotation suggested clear differences between the core and strain-specific gene repertoires. Core gene clusters were primarily associated with housekeeping functions required for bacterial survival, including two-component signal transduction systems, ABC/MFS transporters, central carbon metabolism and cell division. Strain CR191^T^ contained 1014 gene clusters that were absent from the four reference genomes included in the analysis. Functional annotation predicted that some of these strain-specific gene clusters may be associated with osmotic stress responses, *β*-lactam resistance, polysaccharide degradation and interbacterial interactions. In addition, strain CR191^T^ possessed genes encoding LysR/HxlR-family transcriptional regulators, histidine kinases and TonB-dependent receptors, which may be involved in environmental sensing and nutrient acquisition.

Functional annotation of the strain CR191^T^ genome assigned 4369 genes to 26 Clusters of Orthologous Groups (COG) categories (Figure S1). KEGG pathway analysis indicated that 3205 genes are involved in various metabolic processes, with the majority classified under metabolism (74.8%), followed by environmental information processing (11.5%), cellular processes (7.7%), genetic information processing (5.7%), and organismal systems (0.3%) (Figure S2).

A total of 139 genes encoding carbohydrate-active enzymes (CAZymes) were identified and classified into six families: glycoside hydrolase (GH, 52.5%), glycosyltransferase (GT, 33.1%), auxiliary activities (AA, 6.5%), carbohydrate esterase (CE, 5.8%), polysaccharide lyase (PL, 1.4%), and carbohydrate-binding module (CBM, 0.7%). The predominance of glycoside hydrolases suggests a substantial genetic potential for carbohydrate utilization, which may be relevant to its adaptation to carbohydrate-rich environments. Among the 73 glycoside hydrolase (GH) genes identified in the genome of strain CR191^T^, the most abundant families include GH3 (*β*-glucosidases and xylanases), GH5 (endoglucanases and mannanases), GH13 (*α*-amylases and pullulanases), and GH43 (xylanases and arabinofuranosidases), along with members of GH9, GH28, and GH31. These enzymes are implicated in the degradation of plant-derived polysaccharides such as cellulose, hemicellulose, and starch, which are abundant in the maize rhizosphere. The presence of genes encoding both endo-acting and exo-acting glycoside hydrolases indicates a diverse genetic repertoire for carbohydrate degradation and utilization. These genomic features may contribute to the metabolic versatility of strain CR191^T^ in carbohydrate-rich environments.

Genome mining using antiSMASH 6.0.0 identified six putative BGCs associated with secondary metabolism, including two arylpolyene, one NRPS-like, one RiPP-like, one NRPS, and one T3PKS cluster. Several clusters showed similarity to BGCs associated with caryoynencin, malleobactin A/B/C/D, saframycin A/B, cosmomycin D, and xanthomonadin I biosynthesis (Table S2). These findings indicate the biosynthetic potential of strain CR191^T^ for diverse secondary metabolites.

Genomic analysis further identified genes associated with several metabolic pathways in strain CR191^T^, including carbon fixation (ko00720) and the pentose phosphate pathway (ko00030). The genes *accA*, *accB*, *accC*, and *accD*, encoding acetyl-CoA carboxylase, were detected in the genome. Genes associated with the pentose phosphate pathway (e.g., *pgi*, *gnd*, *rpiA*, *rpe*, *tktA*, *tal*, *scpC*, *phaJ*, *fadA* and *atoB*) were also detected, indicating the presence of genes involved in pentose phosphate metabolism. The overall genomic architecture is summarized in a whole-genome circle map (Figure S3).

In addition, screening against the Comprehensive Antibiotic Resistance Database (CARD) identified five antibiotic resistance genes (*rsmA*, *adeF*, *facT*, *adeF*, and *VCC-1*). These genes are predicted to confer resistance to multiple antibiotic classes, including fluoroquinolones, diaminopyrimidines, phenicols, tetracyclines, elfamycins, monobactams, carbapenems, and penams, primarily through mechanisms of antibiotic efflux and enzymatic inactivation.

### 3.3. Morphological, Physiological, and Chemotaxonomic Characteristics

**Figure 5:**
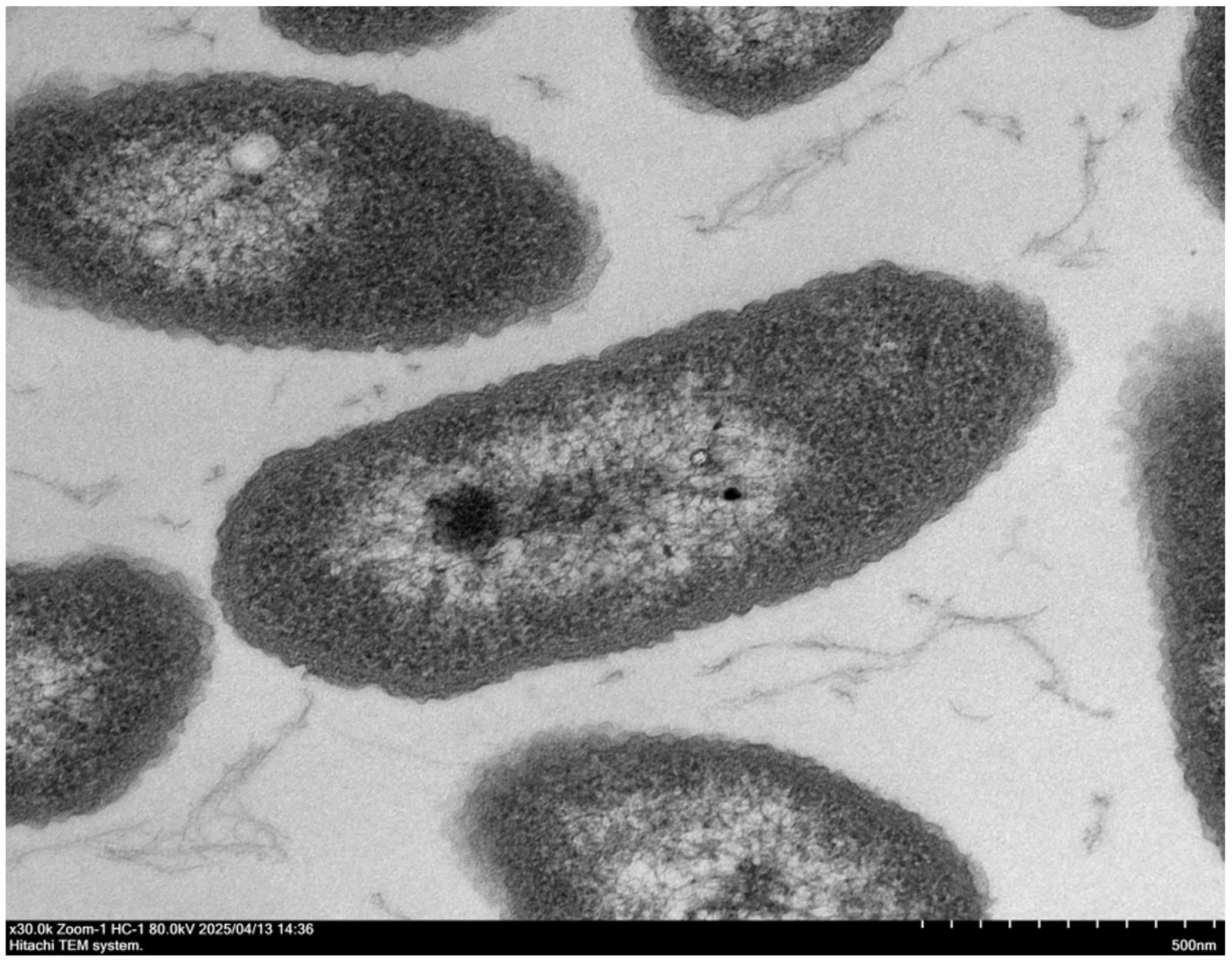
Transmission electron micrograph of strain CR191^T^ after growing on R2A agar medium for 2 days at 30 °C.

Cells of strain CR191^T^ are rod-shaped, Gram-stain-negative, aerobic and non-motile (Figure 5). Key phenotypic and biochemical properties, including enzyme activities and carbon source utilization patterns, are summarized in comparison with related *Dyella* species in Table 1; negative test results are listed in Table S3.

The predominant fatty acids (>10%) of CR191^T^ included C_17:0_ iso (26.1%), iso-C_17:1_ *ω9c* and/or C_16:0_ 10-methyl (summed feature 9, 15.5%), and C_15:0_ iso (12.3%). The cellular fatty acid profile was generally similar to those of other *Dyella* reference type strains (Table S4). Compared with *D. flava* DHOC52^T^, strain CR191^T^ contained lower proportions of anteiso-C_15:0_ and anteiso-C_17:0_, whereas it exhibited higher C_17:0_ iso but lower C_11:0_ iso 3-OH and C_13:0_ iso than *D. dinghuensis* DHOA06^T^. These quantitative differences, which substantially exceeded experimental variability, provided reliable chemotaxonomic markers for distinguishing strain CR191^T^ from its close relatives.

The polar lipid profile of strain CR191^T^, as resolved by two-dimensional thin-layer chromatography using four staining reagents (ninhydrin for aminolipids, molybdenum blue for phospholipids, D reagent for aminolipids, and phosphomolybdic acid for total lipids), consisted of phosphatidylglycerol (PG), phosphatidylethanolamine (PE), phosphatidylmonomethylethanolamine (PME), and two unidentified polar lipids (L1–L2) (Figure S4). The presence of PME distinguished strain CR191^T^ from *D. flava* DHOC52^T^ and *D. nitratireducens* DHG59^T^ [33,34], whereas the absence of diphosphatidylglycerol (DPG) differentiated it from *D. dinghuensis* DHOA06^T^ [35]. The sole respiratory quinone was ubiquinone Q-8 (100%), consistent with other *Dyella* species. Additionally, strain CR191^T^ showed relatively small inhibition zones for several antibiotics, including cefoxitin, tetracycline, clindamycin, penicillin G, vancomycin, and ampicillin (Table S5).

To correlate the genomic predictions with phenotypic susceptibility, the antibiotic resistance genes identified in the genome of strain CR191^T^ were compared with the results of disk diffusion assays. The genome harbors *adeF*, which is associated with multidrug efflux and resistance to several antibiotic classes, including fluoroquinolones and tetracyclines, and VCC-1, an Ambler class A serine *β*-lactamase with carbapenemase activity. However, the presence of these resistance determinants does not necessarily indicate a corresponding resistance phenotype under the tested conditions. These observations highlight the complexity of predicting resistance phenotypes from genotypes and underscore the necessity of experimental validation.

## 4. Conclusions

Comprehensive phylogenetic, phenotypic, chemotaxonomic and genomic analyses consistently demonstrated that strain CR191^T^ represents a novel species of the genus *Dyella*. Beyond taxonomic novelty, genome mining identified six putative BGCs in strain CR191^T^, including clusters showing similarity to known BGCs associated with saframycin, cosmomycin, malleobactin, caryoynencin, and arylpolyene biosynthesis. These findings indicate diverse biosynthetic potential in strain CR191^T^, although the production of the corresponding metabolites remains to be experimentally verified. The predominance of glycoside hydrolases and the presence of strain-specific genes involved in osmotic stress response, polysaccharide degradation, and environmental sensing provide genomic insights into its metabolic potential and possible adaptation to the maize rhizosphere. Collectively, these findings establish *D. cornirhiza* as a novel taxonomic member of the genus *Dyella* and provide a genomic basis for further investigation of its secondary metabolite biosynthetic potential and ecological characteristics. Future studies should focus on experimental characterization of the predicted BGCs and validation of the ecological functions of strain CR191^T^ under biologically relevant conditions.

## 5. Description of *Dyella cornirhiza* sp. nov.

*Dyella cornirhiza* (cor.ni.rhi′za. N.L. fem. n. cornirhiza, referring to the rhizosphere of maize (*Zea mays*), from which the type strain was isolated).

Cells are rod-shaped (0.4–0.6 × 1.5–2.5 µm), Gram-stain-negative, non-motile, and aerobic. Colonies on TSA are smooth, yellow, opaque, circular with entire margins, reaching 1–1.5 mm in diameter after 5 days at 30 °C. Growth occurs at 15–37 °C (optimum, 30 °C), with 0–3.0% (*w/v*) NaCl (optimum, 0%) and at pH 4.0–8.0 (optimum, pH 6.0). The strain is positive for oxidase and catalase activities, but negative for indole and H_2_S production. Aerobic nitrate reduction to nitrite is positive, whereas reduction to nitrogen is negative. The following carbon sources are utilized: dextrin, D-maltose, *β*-formyl-D-glucoside, N-acetyl-D-glucosamine, sodium lactate, fusaric acid, L-glutamic acid, *α*-ketoglutaric acid, L-malic acid, *β*-hydroxy-D, L-butyric acid, D-galactose and D-gentiobiose. The strain weakly utilizes L-arabinose, D-mannose, and L-aspartic acid. The strain is positive for alkaline phosphatase, leucine arylamidase, valine arylamidase, acid phosphatase and naphthol-AS-BI-phosphohydrolase activities. It is positive for esculin hydrolysis and *β*-galactosidase activity. The predominant fatty acids are C_17:0_ iso, iso-C_17:1_ *ω9c* and/or C_16:0_ 10-methyl (summed feature 9), and C_15:0_ iso. The polar lipids include PG, PE, PME, and two unidentified polar lipids. The sole respiratory quinone is ubiquinone Q-8. The DNA G+C content is 66.19%; the genome is 5.21 Mb with 4535 predicted protein-coding sequences (CDS).

The type strain, CR191^T^ (=CCAM 2032^T^ = JCM 36807^T^), was isolated from the rhizosphere soil of maize. The GenBank/EMBL/DDBJ accession numbers for the 16S rRNA gene sequence and the whole-genome sequence of strain CR191^T^ are PV174438 and CP185986, respectively.

## Figures and Tables

**Table 1:** Differential phenotypic and biochemical characteristics of CR191^T^ and the type strains of closely related *Dyella* species.

Characteristic	1	2	3	4	5
Catalase/oxidase	+/+	+/+	+/+	−/+	+/w
Motility	−	−	−	−	−
Temperature for growth (°C)	15–37	10–42	12–37	10–42	10–37
pH for growth	4.0–8.0	4.0–7.0	4.5–7.0	4.0–7.0	6.8–7.5
NaCl range for growth (%, *w/v*)	0–3	0–2	0–1	0–3	0–4
Hydrolysis of:					
Starch	−	+	−	−	−
Arginine	−	+	+	+	−
Urease	−	+	+	+	+
Gelatin	+	−	−	−	+
Enzymic activities					
Esterase (C4)	−	−	w	+	+
Esterase(C8)	−	−	+	+	+
*α*—Galactosidase	−	−	+	+	−
*α*—Mannosidase	−	+	+	+	−
*α*—Fucosidase	−	−	+	+	+
*β*—Glucosidase	−	+	+	+	+
Utilization of:					
D-Fucose	−	+	−	−	+
D—Arabinose	−	−	−	−	+
Amygdalin	−	−	+	−	+
Arbutin	−	−	+	−	+
Cellobiose	−	−	+	+	+
Melibiose	−	−	−	+	+
Sucrose	−	−	−	+	+
Raffinose	−	−	−	+	+
Glycogen	−	−	−	−	+
D—Gentiobiose	+	−	−	+	+
D—Lyxose	−	−	−	+	+
L—Arabinose	w	+	−	+	−
Methyl -*α* -D- glucopyranoside	−	+	+	−	+
DNA G+C content (%)	66.19 †	60.67 †	59.10 †	58.80 †	63.80 †

Strains: 1, CR191^T^; 2, *D. flava* DHOC52^T^; 3, *D. dinghuensis* DHOA06^T^; 4, *D. nitratireducens* DHG59^T^; 5, *D. koreensis* BB4^T^ (data from An et al., 2005 [7]). Symbols: +, positive; −, negative; w, weakly positive; † Data obtained from whole-genome sequences.

## Data Availability

The GenBank/ENA/DDBJ accession numbers for the 16S rRNA gene sequence and the whole genome sequence of strain CR191^T^ are PV174438 and CP185986, respectively.

## Supplementary Materials

The following supporting information can be downloaded at: https://www.mdpi.com/article/10.3390/microorganisms14091943/s1, Figure S1: Number of genes associated with general COG functional categories in the genome of strain CR191^T^; Figure S2: Number of genes associated with KEGG metabolic pathway in the genome of strain CR191^T^; Figure S3: Circular genome map of *Dyella cornirhiza* CR191^T^; Figure S4: Polar lipid profile of strain CR191^T^ based on two-dimensional thin-layer chromatography; Table S1: Comparison of genomic features of strain CR191^T^ and closely related *Dyella* species; Table S2: The prediction of secondary metabolite gene clusters of strain CR191^T^; Table S3: The negative properties of API 20NE, API ZYM, API 50CH and BIOLOG GEN III MicroPlate tests of strain CR191^T^; Table S4: Cellular fatty acid composition (% of totals) of strain CR191^T^ and closely related members of the genus *Dyella*; Excel S1: ANI/dDDH values for 37 species of *Dyella*; Table S5: Antibiotic susceptibility of strain CR191^T^ determined by the disk-diffusion assay.

## Abbreviations

The following abbreviations are used in this manuscript:

KEGG	Kyoto Encyclopedia of Genes and Genomes
ANI	average nucleotide identity
dDDH	digital DNA-DNA hybridization
