# Supplementary material for: Dyella cornirhiza sp. nov., a Maize Rhizosphere Bacterium with Diverse Biosynthetic Potential
Source: Microorganisms. 2026 Sep 2;14(9):1943. doi: 10.3390/microorganisms14091943 (PMC13609492; doi:10.3390/microorganisms14091943)
Supplement: Supplementary file 1 [file microorganisms-14-01943-s001.zip › Dyella cornirhiza-Supplementary Materials.pdf]

Supplementary Materials

Dyella cornirhiza sp. nov., a Maize Rhizosphere Bacterium with Diverse Biosynthetic Potential

Ying Li [1,2], Lei Shen [3], Miaomiao An [1], Xuming Wang [2], Guozhu Zhao [1,*] and Tianlei Qiu [2,*]

[1] National Engineering Research Center of Tree Breeding and Ecological Restoration, College of Biological Sciences and Technology, Beijing Forestry University, Beijing 100083, China; ly19833830219@163.com (Y.L.); anmiao315418@163.com (M.A.)

[2] Beijing Key Laboratory of Agricultural Genetic Resources and Biotechnology, Institute of Biotechnology, Beijing Academy of Agriculture and Forestry Sciences, Beijing 100097, China; wangxuming@baafs.net.cn

[3] College of Life Sciences, Langfang Normal University, Langfang 065000, China; shenlei@lfnu.edu.cn

* Correspondence: zhaogz@bjfu.edu.cn (G.Z.); qiutianlei@baafs.net.cn (T.Q.)

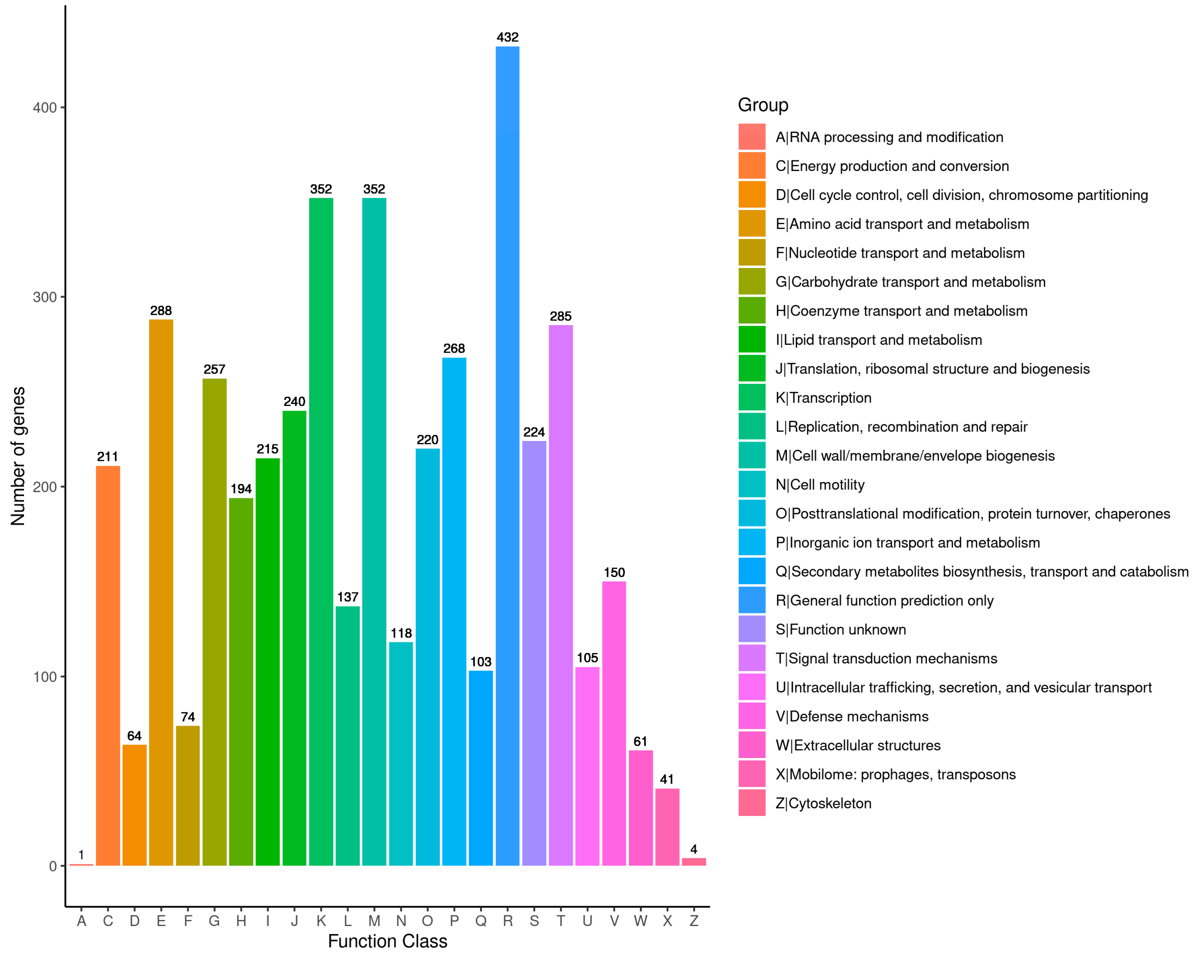

Figure. S1. Number of genes associated with general COG functional categories in the genome of strain CR191^{T}.

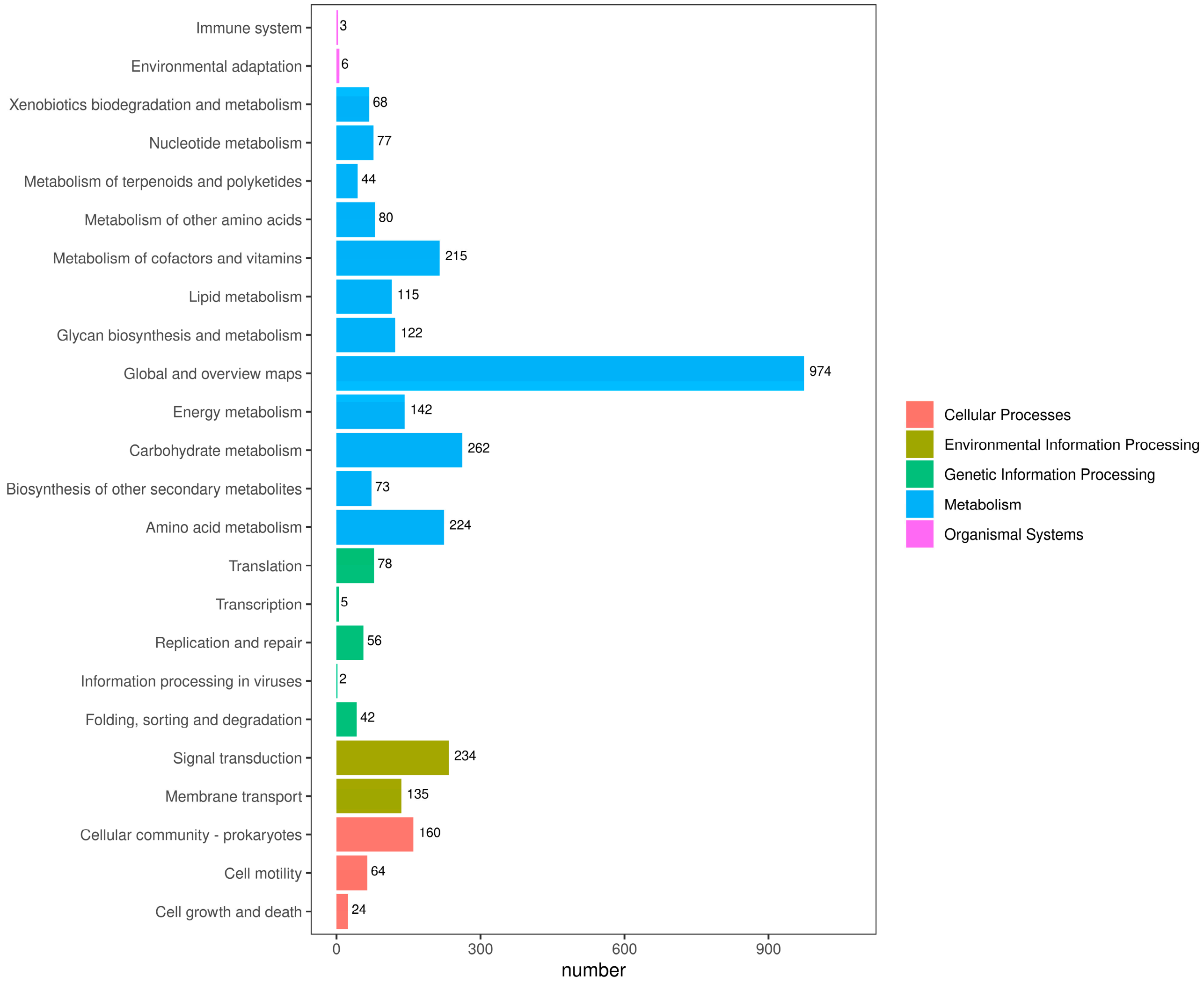

Figure. S2. Number of genes associated with KEGG metabolic pathway in the genome of strain CR191^T.

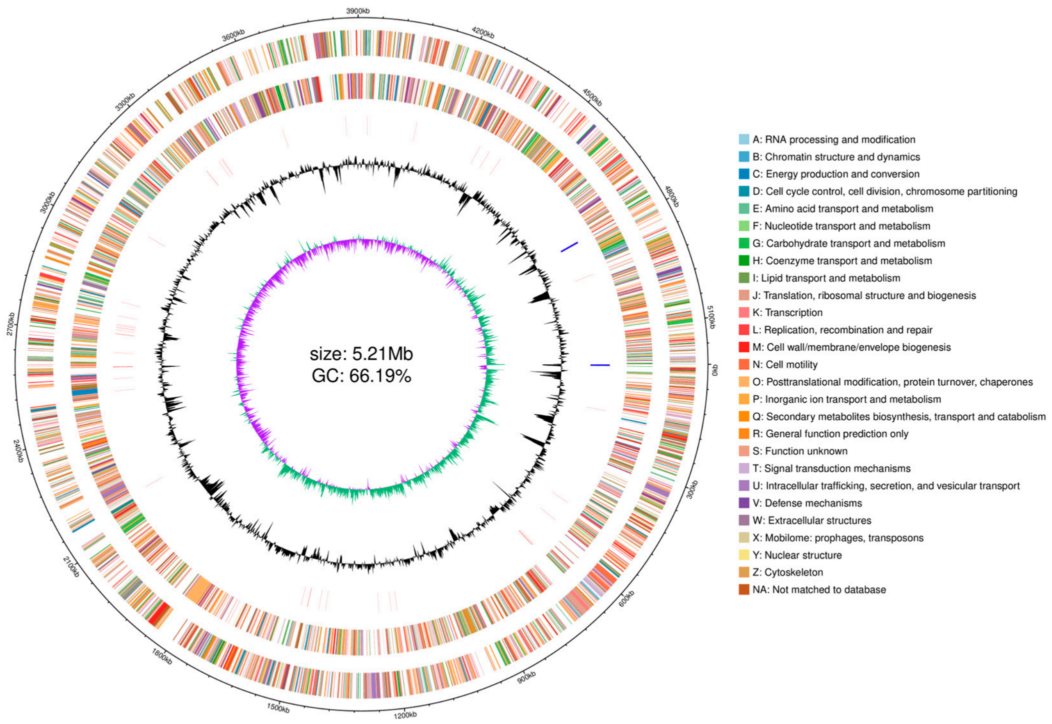

Figure. S3. Circular genome map of *Dyella cornirhiza* CR191T.

From outer to inner: circle 1, scale marker (Mb); circles 2 and 3, protein-coding genes on the forward and reverse strands, respectively, colored by COG functional categories; circle 4, rRNA (blue) and tRNA (red) loci; circle 5, GC content (2000-bp sliding window); circle 6, GC skew (2000-bp sliding window, green: G > C, purple: G < C).

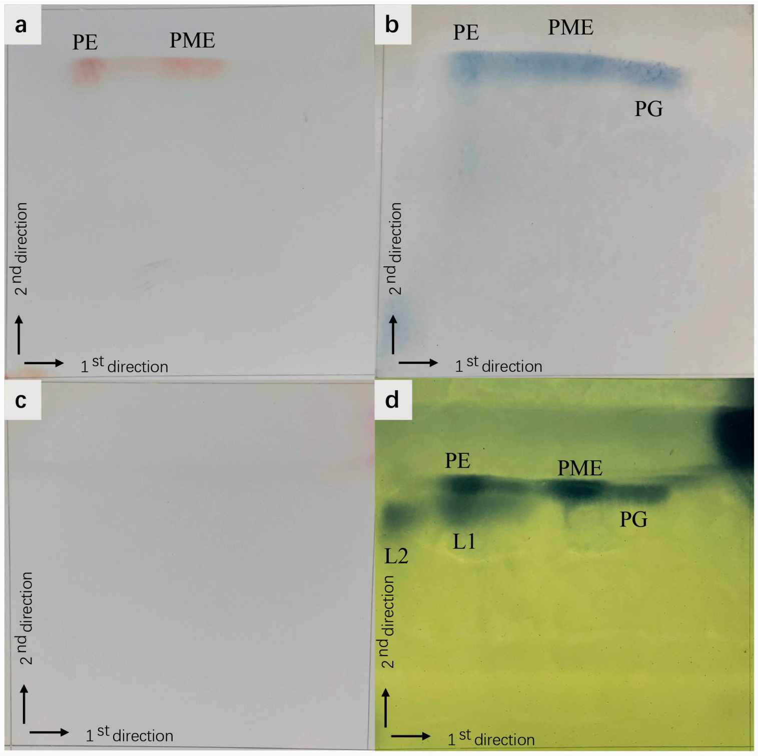

Figure. S4. Polar lipid profile of strain CR191^T based on two-dimensional thin-layer chromatography. Plates were stained with (a) ninhydrin for aminolipids, (b) ninhydrin followed by molybdenum blue for aminophospholipids, (c) D reagent for aminolipids, and (d) phosphomolybdic acid (PMA) for total polar lipids. PG, phosphatidylglycerol; PE, phosphatidylethanolamine; PME, phosphatidylmonomethylethanolamine; L1-L2, unidentified polar lipid.

Table S1. Comparison of genomic features of strain CR191^T and closely related *Dyella* species.

Strains: 1, CR191^T; 2, *D. flava* DHOC52^T; 3, *D. dinghuensis* DHOA06^T; 4, *D. nitratireducens* DHG59^T; 5, *D. koreensis* BB4^T.

**Features**	**1**	**2**	**3**	**4**	**5**
Size (bp)	5,210,872	4,748,678	4,218,720	5,681,744	5,049,380
G+C content (mol%)	66.19	60.67	59.10	58.80	63.80
Number of N50	18,779	386,252	764,301	1,012,417	577,797
Sequencing depth	333×	892×	181×	141×	840×
Total Genes	4,615	4,230	3,748	4,856	4,358
Protein coding genes	4,535	4,146	3,670	4,798	4,280
RNA genes	80	84	78	58	78
GenBank accession no.	CP185986	JADIKE000000000	RYZR00000000	BMJA00000000	JADIKD000000000
ANI (related to CR191^T)	/	80.0%	79.0%	79.6%	83.1%
dDDH (related to CR191^T)	/	21.1%	20.2%	20.6%	23.8%

Table S2. The prediction of secondary metabolite gene clusters of strain CR191^T.

Strains: 1, CR191^T; 2, *D. flava* DHOC52^T; 3, *D. dinghuensis* DHOA06^T; 4, *D. nitratireducens* DHG59^T; 5, *D. koreensis* BB4^T.

Predicted Compound	Synthetase Type	Genes	Size(Kb)
APE Vf	arylpolyene	*thlA, acdA, ydhF, fabG_1, COQ5_1, acpP_2, fabB, fabF_2, arnC_1, acpM, yhdG_2*	46.3
caryoynencin	NRPS-like	*dpp5_1, yfcG_1, lgrD*	41.4
malleobactin A/ B/C /D	RiPP-like	*dhmA_3, bacG_1, pytH_3*	8.4
saframycin A/ B	NRPS	*tylN, dhbF, rimK*	44
cosmomycin D	T3PKS	*ycdF, dap_9, pdxI, hcaB_1, ctaB*	41.1
xanthomonadin I	arylpolyene	*truC, aceA, gpmA, ppm1, acpP_5, glmU*	41.2

Table S3. The negative properties of API 20NE, API ZYM, API 50CH and BIOLOG GEN III MicroPlate tests of strain CR191^{T}.

**Characteristic**
**API 20NE tests:**
Indole production, glucose, arginine dihydrolase, urease, potassium gluconate, D-mannitol, trisodium citrate, phenylacetic acid, capric acid, adipic acid
**API ZYM tests:**
Esterase (C 4), lipase (C 8), lipase (C 14), cystine arylamidase, trypsin, chymotrypsin, α-galactosidase, β-glucuronidase, α-glucosidase, β-glucosidase, N-acetyl-β-glucosaminidase, α-mannosidase, α-fucosidase
**API 50CH tests:**
Erythritol, D-ribose, methyl-β-D-xylopyranoside, xylose, L-sorbose, L-rhamnose, galactitol, methyl-α-D-mannoside, Methyl-α-D-glucopyranoside, amygdalin, arbutin, D-lactose, D-cellobiose, D-salicin, melibiose, D-Sucrose, D-trehalose, inulin, D-pinitol, starch, glycogen, xylitol, D-arabinose, D-pinose, D-lyxose, D-tagatose, arabitol, potassium 2-ketogluconate, potassium 5-ketogluconate
**BIOLOG GEN III MicroPlate tests:**
Stachyose, melezitose, raffinose, N-acetyl-β-D-mannosamine, N-acetyl-D-galactosamine, N-acetylneuraminic acid, D-fructose, 3-formylglucose, fucose, inosine, D-serine, D-sorbitol, inositol, glycerol, D-glucose-6-PO_4, D-fructose-6-PO_4, D-aspartic acid, cetylglutamic acid, L-alanine, L-arginine, L-histidine, L-pyroglutamic acid, L-serine, sodium tetradecyl sulfate, pectin, D-galacturonic acid, L-galacturonic acid lactone, D-gluconic acid, D-glucuronic acid, glucuronamide, mucic acid, saccharic acid, quinic acid, p-hydroxyphenylacetic acid, O-methyl pyruvate, methyl D-lactate, L-lactic acid, citric acid, bromosuccinic acid, lithium chloride, potassium tellurite, tween 40, γ-aminobutyric acid, α-hydroxybutyric acid, α-ketobutyric acid, acetoacetic acid, propionic acid, acetic acid, formic acid, sodium butyrate, sodium bromate

Table S4. Cellular fatty acid composition (% of totals) of strain CR191^T and closely related members of the genus *Dyella*.

Strains: 1, CR191^T; 2, *D. flava* DHOC52^T; 3, *D. dinghuensis* DHOA06^T, 4, *D. nitratireducens* DHG59^T. Major components (>10%) are highlighted in bold.

**Fatty acid***	**1**	**2**	**3**	**4**
$C_{11:0}$ iso	4.84	-	1.55	3.8
$C_{11:0}$ iso 3OH	3.81	-	**11.68**	5.48
$C_{12:0}$ iso	-	-	1.55	-
$C_{13:0}$ iso	-	-	**11.68**	-
$C_{13:0}$ anteiso	-	-	3.55	-
$C_{13:1}$ at 12-13	-	-	2.6	-
$C_{13:0}$ iso 3OH	2.91	-	-	3.75
$C_{14:0}$ iso	-	2.72	5.17	-
$C_{14:0}$	-	1.45	2.6	-
$C_{15:0}$ iso	**12.34**	**17.88**	**14.28**	**31.91**
$C_{15:0}$ anteiso	3.69	**41.76**	7.49	3.11
$C_{16:0}$ iso	5.91	5.52	6.69	3.29
$C_{16:0}$	9.11	7.9	**22.55**	3.63
$C_{16:1}$ *w9c*	-	-	-	-
$C_{17:0}$ iso	**26.14**	2.45	6.66	**12.09**
$C_{17:0}$ iso 3OH	-	-	-	1
$C_{17:0}$ anteiso	3.82	**13.89**	2.78	-
$C_{17:1}$ iso *w10c*	-	-	1.02	-
$C_{18:0}$	-	1.77	1.93	-
$C_{18:0}$ iso	1.01	-	-	-
$C_{20:0}$	1.39	-	-	-
Summed Feature 3†	3.04	-	3.53	5.44
Summed Feature 4†	1.46	2.01	2.3	-
Summed Feature 9†	**15.53**	-	-	**17.73**

*All data were obtained in this study.

†Summed features are fatty acids that cannot be resolved reliably from another fatty acid using the chromatographic conditions chosen. The MIDI system groups these fatty acids together as one feature with a single percentage of the total. Summed feature 3 including $C_{16:1}$ *w7c* and/or $C_{16:1}$ *w*6c; Summed feature 4 including $C_{17:1}$ iso I/anteiso B; Summed feature 9 including iso-$C_{17:1}$ *w9c* and/or $C_{16:0}$ 10-methyl.

Table S5. Antibiotic susceptibility of strain CR191^T determined by the disk-diffusion assay. Inhibition zone diameters are presented as mean ± SD (n = 3).

Antibiotic	Disk content	Inhibition zone diameter (mm)
Norfloxacin	10 μg	18.0±0.2
Carbenicillin	100 μg	22.2±0.3
Cefoxitin	30 μg	6.0±0.2
Streptomycin	300 μg	19.2±0.3
Tetracycline	30 μg	6.0±0.1
Clindamycin	2 μg	6.0±0.2
Levofloxacin	5 μg	24.3±0.1
Erythromycin	15 μg	22.2±0.1
Gentamicin	10 μg	18.3±0.3
Trimethoprim/sulfamethoxazole	25 μg	22.0±0.3
Penicillin G	10 IU	6.0±0.1
Vancomycin	30 μg	6.0±0.1
Neomycin	30 μg	17.0±0.4
Amikacin	30 μg	18.5±0.2
Kanamycin	30 μg	22.2±0.2
Ciprofloxacin	5 μg	22.2±0.4
Ceftriaxone	30 μg	23.2±0.5
Ampicillin	2 μg	6.0±0.2
Cefoperazone	30 μg	18.0±0.2
Enrofloxacin	5 μg	24.0±0.2
Florfenicol	30 μg	24.5±0.4
Polymyxin B	300 IU	18.2±0.2
Rifampicin	5 μg	22.0±0.2
